# Healthy ageing in long-term care? Lessons learned from the COVID-19 pandemic: a position paper

**DOI:** 10.1017/S1463423624000598

**Published:** 2024-11-29

**Authors:** Frode F. Jacobsen, Stinne Glasdam, Heidi Haukelien, Maria E.T.C. van den Muijsenbergh, Gudmund Ågotnes

**Affiliations:** 1 Centre for Care Research, Western Norway, Western Norway University of Applied Services, Bergen, Norway; 2 VID Specialized University, Bergen, Norway; 3 Department of Health Sciences, Faculty of Medicine, Lund University, Lund, Sweden; 4 Centre for Care Research, University of Southeastern Norway (USN), Notodden, Norway; 5 Telemark Research Institute, Bø, Norway; 6 Radboud University Medical Centre, Nijmegen, The Netherlands; 7 Pharos, National Centre of Expertise on Health Disparities, Utrecht, The Netherlands; 8 Department of Welfare and Participation, Western Norway University of Applied Sciences, Bergen, Norway

**Keywords:** Healthy ageing, older adults, long-term care, pandemic, safety, meaningfulness, staffing, built environment

## Abstract

**Aim:**

This position paper focuses on healthy ageing for the frailest and institutionalized older adults in the context of the recent pandemic. The paper aims to identify and discuss hindering and promoting factors for healthy ageing in this context, taking both health safety and a meaningful social life into account, in a pandemic situation and beyond.

**Background:**

The recent COVID-19 pandemic has highlighted the vulnerability of frail older adults residing in long-term care institutions. This is a segment of the older population that does not seem to align well with the recent policy trend of healthy and active ageing. The need for healthy ageing in this population has been voiced by professionals and interest organizations alike, alluding to inadequate support systems during the pandemic, conditioned by both previous and newly emerging contextual factors. Supporting healthy ageing in older adults in nursing homes and other residential care settings calls for attending to meaningful social life as well as to disease control.

**Methods:**

Findings and early conclusions leading up to the position paper were presented with peer discussions involving healthcare professionals and researchers at two joint EFPC PRIMORE workshops 2021 and 2022, as well as other international research seminars on long-term care. The following aspects of long-term care and COVID-19 were systematically discussed in those events, with reference to relevant research literature: 1. Long-term care policies, 2. pre-COVID state of long-term care facilities and vulnerability to the pandemic, 3. factors influencing the extent of spread of infection in long-term care facilities, and 4. the challenge of balancing between strict measures for infection control and maintaining a meaningful social life for residents and their significant others.

**Findings:**

A policy shift towards ageing at home and supporting the healthiest of older adults seems to have had unwarranted effects both for frail older adults, their significant others, and professional care staff attending to their needs. Resulting insufficient investment in primary health care staff and in the built environment for frail older adults in nursing homes were detrimental both for the older adults living in nursing homes, their significant others, and staff. More investment in staff and in physical surroundings might improve the quality of care and the social life of older adults in nursing homes in a non-pandemic situation and be a resource for primary health care staff ensuring both protection from health hazards and a meaningful social life for frail older adults in a pandemic or epidemic situation. As for investing in the physical surroundings, smaller nursing homes are advantageous, with singular resident rooms and for developing out-and indoor spaces for socializing and for meeting with families and other visitors. Regarding investment in staff, there is a documented need for educated staff in full-time positions. Use of part-time or temporary staff should be limited.

## Background

In both policy documents and research literature, healthy ageing is frequently related to how an ageing population increasingly could and should remain active in later life. Active and healthy ageing was proposed by the World Health Organization (WHO 2020), who defines healthy ageing as ‘the process of optimizing opportunities for health and enhance quality of live as people age [applying to] both individuals and groups’ (Bousquet *et al.* 2015:956). The related concept of active ageing is defined by WHO as ‘the process of optimising the opportunities for health, participation, and security in order to enhance quality of life in older age’ (Nordic Welfare Centre, 2022). Relatedly, an Active Ageing Index (AAI) has been developed as a tool to support the implementation of so-called social investment orientation in policies. This aims to leverage the potential of older people to contribute to society. The AAI comprises 22 individual indicators across four domains: employment, participation in society, independent, healthy and secure living, and capacity and enabling environment for active ageing (European Commission, 2021).

While the recent policy trend of active ageing entails a positive view on ageing, in which the potential for continuous personal development and living an active and meaningful life in older age are stressed, the frailer part of the older population appears to have received less attention in health policies (Dahl, 2017; Gibbons, 2016; Munkejord *et al.*, 2018). This position paper focuses on opportunities and obstacles for healthy ageing of the frailest older adults in the context of the COVID-19 pandemic, namely, older adults in nursing homes and other long-term care institutions within the in primary health care system.

With increased longevity worldwide, where the share of people 60+ is higher than the share of children younger than five for the first time and is expected to double by 2050. Consequently, the number of older people experiencing frailty is steeply increasing (WHO, 2022). Old age frailty is characterized by increased vulnerability due to a clinically recognizable decline across multiple physiological systems. This results in slower recovery from illness and other adverse events and a reduced ability to cope with everyday life. Frailty indicators include low energy, low physical activity, slow walking speed, low grip strength, and unintentional weight loss (Fried *et al.*, 2001).

### Developments in long-term care across European countries

In line with the general population ageing across European countries, expenditure on long-term care (LTC) has been rising faster than that on general health care (Costa-Front, Courbage and Zweifel, 2017). However, compared with other welfare services in most European countries, LTC still seems to be substantially less subsidized (Costa-Font and Zigante, 2020). The growth in public expenditure for LTC services is expected to continue to increase (Barczyk and Kredler, 2019), a trend that is not merely propelled by the population ageing, but also other factors such as changing family structures and an increase in female working life participation (Costa-Font and Zigante, 2020).

The cultural and historical context for design and organization of long-term care appear to be important (World Bank, 2010). While public home-based care in Europe is a post WWII phenomenon, institution-based care, mainly nursing homes, has longer historical roots, originally deriving from the so-called poor house around the mid-19^th^ century. For several decades, until the late 1970ies and early 1980ies, the hospital used to be the ideal model for architects and planners of nursing homes. While the coverage of nursing homes and their form and content vary much across Europe, a general trend of profiling nursing homes as ‘real’ homes has taken place in most of Europe since around mid-1980ies. Smaller residential units and singular occupancies with room for own furniture and personal belongings have many places been part of this trend (Hauge and Heggen, 2008).

How formal long-term care is organized varies much between European countries. A study investigating the organization of long-term care in ten old and 11 new EU members states found that in around half of the countries, the regulation and decision-making responsibility was at the national level, while responsibility was shared across different government levels in the other half. This holds true both for institution-based and homebased care. In general, regulation and organization of care seem to be more centralized in new member states, like, for example, the Czech Republic and Romania, compared to old member states, like for example, France and the Netherlands (Riedel, Kraus and Mayer, 2016). Nearly all of the 21 countries have legal entitlement to long-term care, with the exceptions of England, Austria, and Romania, where, e.g., England entitlement is granted for home nursing but not for institution-based nursing or practical home help. In six of the 21 countries, long-term care is means tested, with no tendency towards geographical clustering or any clear difference as to old and new EU member states (ibid.).

The public expenditure of LTC services across Europe varies substantially between countries, as does informal care from family members. In Europe, the Scandinavian and Benelux countries appear as the most universally publicly funded as to LTC services, where benefits for people in need of LTC services are typically in kind rather than cash benefits (Barzcyk and Kredler, 2019). Southern European countries like Spain and Italy, by contrast, tend to have the smallest LTC public spending, the largest private individual spending, and the least comprehensive formal LTC sector. The central European countries, like Germany, Austria, and France, tend to have a more extensive public funding than southern countries but less than northern European countries. This north-south gradient seems to go together with an inverse trend as to contribution of informal care: informal care plays a significant role in the southern countries as compared with the northern ones. Furthermore, the nursing home coverage and availability is substantially lower in southern than in northern countries (Barzcyk and Kredler, 2019).

As to central and eastern European countries, there seems to be much variation regarding how LTC is covered and as to the availability of nursing homes and other LTC services (van Eenoo *et al.*, 2016; Luczak, 2018). As an example of the variation in the LTC services, nursing homes (sometimes labelled care homes in international literature), seem to be the most common formal care institutions for so-called dependent people (Necel, 2023) and have increased in number and availability since the early 1990ies. By contrast, the Czech Republic has far more developed system of social care than supply of nursing homes (Luczak, 2018). In general, long-term care appears to be primarily delivered in the form of institution-based care (like nursing homes) in new EU member states, whilst home-based care (including home nursing) seems more developed in old EU member states (Riedel, Kraus and Mayer, 2016).

The type of legislation governing long-term care appears to vary much across European countries as well. Even within the Scandinavian countries, the situation is not uniform. For example, long-term residential care is covered by social and housing legislation in countries in Sweden and Denmark while being part of health and medical legislation in Norway (Daatland *et al.*, 2015).

Eurofound’s reports from 2020 and 2022 and the Organisation for Economic Co-operation and Development (OECD’s) report from 2023 highlight critical challenges faced by the long-term care sector. Both the quality of and access to long-term care were negatively affected by the pandemic. The pandemic significantly strained the sector, revealing pre-existing issues such as staff shortages, low wages, high job insecurity, and limited career progression opportunities. Additionally, workers often endure physically and emotionally demanding conditions, long hours, and inadequate working environments, which contribute to high levels of stress and job dissatisfaction. The pandemic exacerbated these problems, leading to increased workloads and insufficient support for many workers (Eurofound 2020; 2022; OECD 2023).

### Active ageing policy trend in Europe: benefiting the frailest old?

In 2012, the European Union stated that ‘never before has Europe enjoyed such a large proportion of healthy older people’ (European Union, 2012:1). The year 2012 was the European Union’s Year for *Active Ageing and Solidarity between Generations*, a commitment that later has been referred to and reflected in European health policy documents. The policies aiming at active and healthy ageing pay attention to the trend that people in Europe and beyond are ageing, and that, even though the number of frail and ill older adults is increasing, the proportion of assumed healthy older adults is growing (WHO, 2022). Considering this, states and public sectors have gradually moved away from a model of providing for, caring for, and protecting the general populations, towards a model of activation and rehabilitation (Torfing, 2004; Pedersen, 2011), in which prevention as opposed to treatment is prioritized, and where self-reliance is emphasized (Stephens, 2017).

Clearly, a policy of healthy ageing, in which older adults’ resources and capabilities are emphasized and where the concept of healthy ageing includes active ageing (WHO 2020), has the potential to benefit many. An ageist approach, by contrast, is characterized by lack of appreciation of skills, potentials, and resources in older adults. Still, the questions can be raised if this recent policy trend is equally beneficial for all segments of a growing ageing population. While relatively healthy older adults may find themselves more included in the general society than before this recent trend, the policies might not be as adapted towards all older people, in particular frail older adults (Munkejord *et al.*, 2018). In other words, discourses addressing resources, potentials, and capabilities of older adults might be exclusionary by default for many of them (Dahl, 2017; Gibbons, 2016; Stephens, 2017). Frail older adults, not the least, older adults living in nursing homes, may hence have become less visible in the context of the ageing at home/ageing in place policy trend. This may have become an unwarranted consequence of shifting the attention towards the healthiest of older adults.

The COVID-19 pandemic has both highlighted and aggravated the vulnerability of the frailest older adults. Internationally, the outbreak of COVID-19 has affected people living in nursing homes particularly hard (Comas-Herrera *et al.*, 2020), influencing their somatic, mental, and social health. Consequently, the pandemic has highlighted dimensions of nursing home care that need to be reflected on and improved, not the least, related to social activities and to supporting social relationships with significant others, relevant also in a non-pandemic situation.

Present unmet needs, loneliness, and deterioration of physical abilities and cognitive functioning in this population are all examples in the research literature of how a vulnerable population became more vulnerable during the pandemic (Lood *et al.*, 2021; Noten *et al.*, 2022; Paananen *et al.*, 2021; Pérez-Rogdrígues *et al.*, 2021; Rand *et al.*, 2022; Van Maurik *et al.*, 2020). Measures of infection control involved harsh means of physical isolation on facility level, ward level, and in many cases resulted in older adults being isolated in their own room or apartment for substantial periods of time.

The OECD (2021) underscored the need for comprehensive and sustained policy efforts to address the challenges faced by the LTC sector, enhance its resilience, and ensure high-quality care for older adults. Also, the European Commission *et al.* (2021) underscored the urgency of addressing the challenges facing LTC systems across Europe and provided valuable insights into potential reforms and improvements that can help ensure high-quality and sustainable care for the ageing population.

The primary questions asked in this position paper are:

1. How can healthy ageing be supported for frail older adults in nursing homes and other long-term residential care settings, in the event of a pandemic/epidemic and in general?
2. What obstacles and opportunities can be identified for aiming at both safety from infectious diseases and meaningful social lives for older adults?

## Aim

The aim of this position paper is to assist primary health care (PHC) providers, policymakers, and researchers by discussing the current context in which health care for frail older patients functions within PHC in Europe, amongst other, by identifying hindering and promoting factors for healthy ageing in the population of older people in long-term residential care in the event of the pandemic and beyond. The position paper seeks to discuss changes in service models, policy, education, and research in the PHC context. A wider examination of home palliative care models and a critical appraisal of the variation in findings will help improve the evidence base for the development, implementation, and evaluation of home palliative care services in the future. Informed by expert opinions and a wide range of research literature, it provides evidence for improvements to patients, families, and health systems.

## Methods

Findings and early conclusions leading up to the position paper were presented with peer discussions involving healthcare professionals and researchers at two joint EFPC PRIMORE workshops 2021 and 2022, as well as other international research seminars on long-term care. The following aspects of long-term care and COVID-19 were systematically discussed in those events, with reference to relevant research literature: 1. Long-term care policies, 2. pre-COVID state of long-term care facilities and vulnerability to the pandemic, 3. factors influencing the extent of spread of infection in long-term care facilities, and 4. the challenge of balancing between strict measures for infection control and maintaining a meaningful social life for residents and their significant others.

Around 20 participants with a diversity of professional and academic backgrounds like sociologists, anthropologists, general practitioners, nurses, and social workers took part in the workshops. No audio recording was performed during the workshops, however, written notes from discussions were taken. The participants consented to sharing the resulting information, where key themes identified from this information were actively used to perform a second round of literature search for this position paper.

Presentations at three international seminars on long-term care and COVID-19, led by London School of Economics (LSE), provided an opportunity for cross-country comparison and for identifying important insights from research literature. Each seminar had between 30 and 40 participants, where the majority had a background in the social sciences (political science, economy, sociology, and anthropology), while also professional like nurses and general practitioners contributed. The cross-country comparison was carried out by utilizing national statistical sources as to, e.g., prevalence of COVID and COVID-related deaths in nursing homes. In addition, a range of scientific publications were utilized where also qualitative dimensions like approaches to LTC and to prevention and relating to incidents of COVID-infections in nursing homes. Based on this material, the present paper discusses the balance between supporting a rich social life, with meaningful activities for the individual, and safety from health hazards, in particular, in pandemic or epidemic situations.

## The argument

The aim of healthy ageing is far from being achieved for the frailest old, the older adults living in nursing homes. A nursing home is both a health institution and a last home for its residents. Hence, staff and social and material surroundings need to support both health and a meaningful social life. In a pandemic situation, sufficient and competent staff, who know how to limit the spread of infection, and a well-designed physical environment appear as vital prerequisites in order to achieve such an aim for this population.

## Findings

The scarce available published international studies concluded that older adults living in nursing homes had experienced severe consequences of the pandemic, leading to social isolation and considerable cognitive decline because of decreased stimulation in daily living (Leskovar and Klemencic 2022; Pérez-Rogdrígues *et al.*, 2021; Rand *et al.*, 2022; Van Maurik *et al.*, 2020). Physical environment and staff-related dimensions appeared to be important factors influencing the extent to which older adults in nursing home may experience healthy ageing.

Internationally, a large share of COVID-19-related deaths took place in nursing homes during the early phase of the pandemic (Comas-Herrera *et al.*, 2020). In Scandinavian countries, for example, the share of COVID-19 deaths in nursing homes was around 50 % during the first year of the pandemic (Jacobsen *et al.*, 2021; Szebehely, 2020). The pandemic hit unevenly, in European countries and beyond, with frequently only a minority of the nursing homes representing the bulk of the infected persons living in nursing homes nationally (Liu *et al.*, 2020; Szebehely, 2020). Like in the instance of Norway, by end of April 2020, after the most severe surge of nursing home infections during the pandemic, only 31 of the 800 Norwegian nursing homes were affected, while some of these institutions had a very high death toll where the 10 institutions most severely hit accounted for 40 % of the total death toll in nursing homes (Jacobsen *et al.*, 2021). There were probably at least two possible explanations for this. Firstly, once COVID-19 entered a nursing home, it was difficult to minimize spread of the infection to other older adults and the staff. Secondly, facility characteristics seemed to play a role, both related to parttime and temporary staff, and to the architecture and physical environment (Abrams *et al.*, 2020; Jacobsen *et al.*, 2021; Sabatino and Harrington, 2021).

### Physical environment

The built environment of nursing homes both influenced the extent to which they avoided or limited infection, and the extent to which they allowed for social activities and social life for people living there. Regarding infection control, the size of nursing homes has been found to be correlated with higher numbers of infected older adults. Large facilities were associated with a significantly higher incident of COVID-19 infections than smaller facilities in both North American (Abrams *et al.*, 2020; Mathematica, 2020; Sabatino and Harrington, 2021) and Australian studies (Ibrahim *et al.*, 2021). In particular, nursing homes that are both large and old have proved vulnerable to outbreaks of COVID-19 (Stall *et al.*, 2020).

By contrast, smaller nursing homes had averagely less COVID-19 infections and deaths than larger nursing homes. This was seen in smaller more home-like nursing homes having singular occupancies with own bathrooms, a stable staff with restricted use of non-permanent and parttime positions, and having cooking and laundry services ‘in-house’ (Zimmerman *et al.*, 2021). Furthermore, nursing homes combining smaller units and singular occupancies with good outdoor areas, including balconies and accessible green open spaces onsite or nearby the nursing home, also had less COVID-19 infections (Leskovar and Klemencic, 2022). Nursing homes combining singular occupancies with smaller units that can be physically closed off in the event of an outbreak and indoor spaces for meetings and social encounters in the nursing home at safe distance from the wards, also seem to limit contagion (Jacobsen *et al.*, 2021). In addition, those types of smaller nursing homes made a combination of infection control and a rich social life possible (Jacobsen *et al.*, 2021; Leskovar and Klemencic, 2022; Zimmerman *et al.*, 2021).

Features such as old and impractical buildings together with lack of access to protective gear and staff members in quarantine have been identified in facilities most severely infected by COVID-19 (Jacobsen *et al.* 2021; Strand 2020). Examples of building features that seem to have played a significant role, in addition to the already mentioned presence or absence of singular occupancies and opportunities for physically closing off singular wards if infections occurred, are enough exclusive staff space for keeping clean clothes and protective gears separate when changing clothes and having floors and walls that were easy to keep clean (Jacobsen *et al.*, 2021).

The built environment of nursing homes could, thus, imply barriers and opportunities for handling a pandemic (or epidemic) situation. How the built environment functioned in total as to keeping older adults safe for infection, and at the same time supporting their social activities and general well-being, related to how it functioned for all the three main categories using the nursing home environment: first and foremost, the older adults living there, but also significant others (Jacobsen *et al.*, 2021) and staff (Duijs et al, 2023; Rutten *et al.*, 2022).

### Staff-related conditions and other contextual conditions

Nursing home size and building characteristics were also interrelated with other influential factors connected to how the COVID-19 situation had been handled. An unfortunate interplay between inadequate built environment and a widespread parttime staff employment, where the same staff members worked in several institutions, seemed to have led to a small fraction of nursing homes being particularly exposed to COVID-19 (Jacobsen *et al.*, 2021). The number of cases of infected older adults and staff the nursing homes had in the different countries seems, to some extent, to reflect the general level of COVID-19 infections in the country (Comas-Herrera *et al.*, 2020). However, why, in many cases, a smaller selection of nursing homes represented a significant proportion of deaths could not be explained by this fact. In addition to size and physical characteristics of nursing homes, parttime staff positions were a factor for spreading the disease (Jacobsen *et al.*, 2021; Szebehely, 2020).

Several studies supported the insight that the conditions for staff were conditions for care. Nursing homes with higher staff-to-resident ratio had less incidents of COVID-19 among the older adults (Liu *et al.*, 2020; Sabatino and Harrington, 2021; Yau *et al.*, 2021). Full-time employment was an important measure for avoiding spread of infections, while widespread use of part-time positions, leading to same staff working at more than one institution, made the institutions vulnerable to COVID-19 and other infections (Jacobsen *et al.*, 2021; Sabatino and Harrington, 2021). Pre-pandemic low staffing level also had dire consequences during the pandemic. As previously pointed out in this paper, for-profit management of nursing homes tend to be linked to a lower level of staffing than non-profit or public management. The pandemic had a severe impact on nursing homes staffs’ health and well-being as well as working practices, and in particular, in nursing homes with a low staffing level (Hanna *et al.*, 2022; Hoedl *et al.*, 2022; Sarabia-Cobo *et al.*, 2021). Reduced mental health and exhaustion in the nursing staff were a pronounced result (Yau *et al.*, 2021). Several other staff-related factors, such as investment in staff training and education were, for example, important in combating spread of infections (Yau *et al.*, 2021). Dedicated areas for staff also limited spread of infections to older adults (Anderson *et al.*, 2020), as did sufficient supply of protective gear (Jacobsen *et al.*, 2021; Yau *et al.*, 2021).

Financing and ownership were other factors that seemed to play a role in how nursing homes performed during the pandemic. Large-scale studies indicated that for-profit management and ownership were associated with a higher level of infection, as found both for the United States (Mathematica, 2020), Canada (Liu *et al.*, 2020), and Australia (Ibrahim *et al.*, 2021). Public or non-profit management, by contrast, were found to be associated with less infections, better financing, more singular occupancies, and better staff coverage (Liu *et al.*, 2020; Mathematica, 2020).

### Social isolation and consequences for older adults

Ensuring both disease control and securing meaningful social life for older adults in nursing homes and other long-term residential care settings was an aim that can, as demonstrated above, be hampered, or supported by the physical environment, staff-related conditions, and other factors like financing and ownership.

Several studies testified to the dire consequences of the pandemic for older adults living in nursing homes in terms of loneliness and reduced wellbeing (Jacobsen *et al.*, 2021; Levere *et al.*, 2021) and increased use of medication, not the least, psychotropic drugs (Campitelli *et al.*, 2021; Stall *et al.*, 2021). This development appeared in parallel to a general health decline (Thompson *et al.*, 2020), particularly a decline in functional status and mental health (Jacobsen *et al.*, 2021), which works contrary to the defined aim of healthy ageing, which WHO defines as “the process of developing and maintaining the functional ability that enables wellbeing in older age” (WHO, 2020).

A retrospective observational study of three Norwegian nursing homes experiencing early and large-scale outbreak of COVID-19 concluded that the infection in all the cases entered through staff (Kittang *et al.*, 2020). Staff seemed the more likely source of infection than significant others and the consequences for the health and well-being of the older adults of social isolation were severe (Kittang *et al.*, 2020). Hence one may question the potential gain of isolating people living in nursing homes from their significant others.

Not only people living in nursing home wards with outbreaks of infection suffered from increased social isolation. Dire consequences of social distancing measures for people living at nursing homes were identified also in nursing homes and specific wards where there were no infected older adults. There were substantially less activities for the older adults also in those places, in particular social activities. Contact with family and significant others was a huge challenge, in particular in wards with no COVID-19, but with neighbouring wards with infections, where understaffing resulted from transferring staff to wards with COVID-19 infections and hence less time for following up on those relations. Consequently, a decline in social, mental, and physical health of older adults in nursing home wards with and without infections was recognized by interviewed physicians and care staff alike (Jacobsen *et al.*, 2021). Studies from several other European countries showed such dire effects as to lack of social activities and maintenance of social connectedness, for example, a Polish nursing home study (Necel, 2023) and an Italian nursing home study where severe cognitive decline and disruption of social life of older adults were reported (Chirico *et al.*, 2023).

Examples of innovative solutions for older adults’ meeting with significant others were identified, like large tents outside the nursing homes or transforming a meeting room used for educational events into meeting rooms catering to older adults. Still, several significant others complained that older adults in nursing homes seemed to have lost hope after a while and decreasingly sought contact (Jacobsen *et al.*, 2021).

In addition to help guiding the most important themes of this paper and help guiding further literature search following the above mentioned EFPC PRIMORE workshops, the experience and knowledge of the workshop participants have been vital both in strengthening the arguments and validating the conclusions. In general, the participants, all of them from European countries, of whom several have been either researching or working in nursing home as practitioners, subscribed to the importance of the identified organizational and physical aspects for ensuring both safety and a meaningful life for nursing home residents.

## Some lessons learned - recommendations

Balancing infection control and supporting activities and a meaningful social life, thus acknowledging that health and healthy aging are about more than disease control, seem both important and challenging There needs to be a strengthening of the nursing homes in general regarding staffing, terms of employment, and the built environment. The fact that some staff work at more than one nursing home or LTC facility, and sometimes also in other sectors (e.g., restaurants), reveals the need for better paid staff with secure and full-time employment terms. Preparing for future pandemics/epidemics means first and foremost improving conditions for care in general in nursing homes.

The size of nursing homes is well documented to play a role in spread of COVID-19. Older adults living at smaller institutions appear to be better protected than when living at larger ones (McGarry *et al.*, 2021). How space is organized inside the institutions plays an important role as well. Having singular occupancies and smaller wards, which are easier to isolate from each other, makes it easier to curb the spread of infections, while absence of anteroom, a “clean” transition room between older adults’ private room and corridor, and long and narrow corridors and walls with rough surfaces difficult to keep clean, appear as major factors making nursing homes more vulnerable to spread of disease.

A balance needs to be struck between infection control and other measures of patient safety, on the one hand, and supporting contact with significant others and sustaining a meaningful social life, on the other hand. Health and well-being of older adults are about far more than disease control and keeping them safe from diseases and death. Healthy ageing for frail older adults also implies supporting social activities and social networks. A lack of the latter has, as pointed out, dire consequences for social, mental, and physical health and for general well-being of the frail older adults in nursing homes (van der Roest *et al.*, 2020).

The pandemic has exposed pre-pandemic vulnerabilities and shortcomings in the LTC sector all over Europe and beyond. The pandemic has highlighted that the aim of healthy ageing is far from being achieved for the frailest old, and in particular, older adults living in nursing homes. In particular, sufficient competent staff, who know how to limit the spread of infection and a well-designed physical environment appear as vital prerequisites in order to achieve such an aim for this population.

By way of preliminary conclusion, there need to be several practical measures put in place:

-Effective workforce planning and retention strategies to address staff shortages and ensuring the sustainability of LTC services (Eurofound, 2020), realizing that the conditions for work are the conditions for care (Canadian Health Coalition, 2023).

-Investment in education and training programs to equip workers with the necessary skills and qualifications (Eurofound, 2020)

-Development of a nursing home architecture both supporting homeliness and social activities and infection control

## Conclusion

A policy shift towards ageing at home and supporting the healthiest of older adults, seems to result in a de-prioritization of residential homes, effecting both older adults, their significant others, and nursing home staff, and in the long run the quality of our welfare societies. Preventing and handling infections requires professional skills, and these skills are becoming scarce due to this de-prioritization (Haukelien, 2021). The consequences of lack of full-time positions in primary health care staff, a heavy workload, poor working conditions, and huge responsibility for those who work with the frailest older adults is revealed in high rates of COVID-19 infections among older adults living in nursing homes. The research findings clearly indicate a correlation between on the one side what is commonly considered to be good working conditions for the staff and good quality living conditions for older adults, and on the other side high level of infection control. Probably, these patterns represent enforcements of patterns seen under other circumstances; namely an ongoing care crisis in the welfare states (Hansen, Dahl and Horn, 2022).

The aim of healthy ageing in the population of older adults in nursing homes and other LTC facilities had been particularly hampered by the COVID-19 pandemic. The recent COVID-19 pandemic has exposed and highlighted the vulnerabilities of older adults living at nursing homes and of the nursing home as care institutions. Lessons learned from the pandemic have therefore the potential to make the societies better prepared for future epidemics and pandemics, and, ensuring sufficient staff coverage and competence, good routines, and a built environment in nursing homes, which support both a rich social life and keep people safe from infections. Moreover, in a non-pandemic situation with no major adverse incidents, this may contribute to making nursing homes better last homes for the frailest older adults, a vital precondition for ensuring healthy ageing in this population.
